# Therapeutic Potential of Glucose Oxidase-Loaded Biogenic Mesoporous Silica Nanoparticles in Ovarian Cancer

**DOI:** 10.3390/ph18071060

**Published:** 2025-07-18

**Authors:** Andrea G. Uriostegui-Pena, Padmavati Sahare, Gabriel Luna-Bárcenas, Sujay Paul

**Affiliations:** 1 School of Engineering and Sciences, Tecnologico de Monterrey, Campus Queretaro, Querétaro 76130, Mexico; 2 Institute of Advanced Materials for Sustainable Manufacturing, Tecnologico de Monterrey, Campus Queretaro, Querétaro 76130, Mexico

**Keywords:** glucose oxidase, nanoformulation, mesoporous silica nanoparticles, ovarian cancer, starvation therapy

## Abstract

**Background/Objectives:** Ovarian cancer (OC) remains one of the most lethal malignancies of the female reproductive system. Glucose oxidase (GOx) has emerged as a potential therapeutic agent in cancer treatment by inducing tumor starvation through glucose depletion. Nonetheless, its clinical application is constrained due to its systemic toxicity, immunogenicity, poor in vivo stability, and short half-life. These challenges can be addressed through nanotechnology; in particular, biogenic mesoporous silica nanoparticles (MSNs) offer promise as drug delivery systems (DDSs) that enhance therapeutic efficacy while minimizing side effects. **Methods:** Biogenic MSNs were extracted from the *Equisetum myriochaetum* plant via acid digestion, functionalized with 3-aminopropiltrietoxysilane (APTES) and glutaraldehyde (GTA), and loaded with GOx. The free and immobilized MSNs were characterized using FTIR, DLS, XRD, SEM/EDX, and BET techniques. A colorimetric approach was employed to quantify the enzymatic activity of both the free and immobilized GOx. The MTT assay was employed to assess the viability of SKOV3 cells. The obtained IC_50_ concentration of the nanoformulation was administered to SKOV3 cells to analyze the expression of cancer-related genes using RT-qPCR. **Results:** IC_50_ values of 60.77 ng/mL and 111.6 µg/mL were ascertained for the free and immobilized GOx, respectively. Moreover, a significant downregulation of the oncogene β-catenin (*CTNNB1*) was detected after 24 h with the nanoformulation. **Conclusions:** Our findings indicate that GOx-loaded biogenic MSNs may serve as a potential therapeutic agent for ovarian cancer. This is, to the best of our knowledge, the first report exploring the effect of GOx-loaded biogenic MSNs on SKOV3 cells.

## 1. Introduction

Ovarian cancer (OC) holds fifth place as the most lethal cancer among women worldwide, with epithelial ovarian cancer (EOC) being the most menacing malignancy of the female reproductive system due to its mortality rate of over 64% [1,2]. The number of cases and deaths resulting from OC are of great concern, being 313,959 and 207,252 in 2020, respectively, with the highest incidence observed in high-income level countries, including Central, Eastern, and Northern Europe; Polynesia; and North America [3]. Although the etiology of OC remains unknown, factors including advanced age, early onset of menarche, late onset of menopause, nulliparity, ethnic background, endometriosis, obesity, smoking, and family history have been identified as factors that increase the risk of developing OC [4,5,6]. It is hypothesized that persistent ovulation, inflammation, and gonadotropin and hormonal stimulation may be critical factors for susceptibility to OC, some related to the damage occurring during ovulation and repair of the ovarian surface epithelium [6,7]. Germline mutations in *BRCA1* and *BRCA2* genes, as well as mutations in mismatch repair genes—including *TP53*, *STK11*, *CHEK2*, *BRIP1*, *PALB2*, and *RAD51*—increase susceptibility to malignancy [4,5]. However, the aberrant expression of several genes has been noted in OC. For instance, *BCL-2* and *BAX* expression serves as a prognostic factor in OC due to their role in apoptotic regulation [8,9,10]. The oncogene *CTNNB1* is overexpressed threefold in OC tissues, and an increase in *WNT1* has been observed in OC compared to normal ovaries [11]. These gene expression alterations reflect some of the underlying molecular mechanisms of the disease, highlighting the importance of identifying specific biomarkers for improved diagnosis and therapeutic targeting.

Despite these molecular insights, the clinical management of OC remains challenging. The delayed diagnosis of OC, primarily due to the lack of early-stage symptoms, leads to poor survival outcomes, especially once the disease has spread beyond the pelvis [12]. Alarmingly, only about 15% of OC cases are diagnosed within stage I, given that progression to advanced stages occurs within a year [2]. Debulking surgery and chemotherapy remain the primary standard treatment for EOC [12,13]. Platinum-based chemotherapy, in which a combination of carboplatin and paclitaxel is used post-surgery, is often used every 3 weeks [13,14]. Other strategies for OC treatment include neoadjuvant therapy, poly (ADP-ribose) polymerase inhibitors, or the use of non-platinum agents such as paclitaxel, pegylated liposomal doxorubicin, or topotecan alone or in combination with bevacizumab [14,15]. Although the detection of OC in early stages translates to a 92% 5-year survival rate, it diminishes to 30% for patients diagnosed in later stages, usually followed by recurrence within 2 years [2,12,16]. Consequently, there is an urgent need to explore and develop new therapeutic strategies that improve clinical outcomes and reduce recurrence rates in OC patients.

Glucose oxidase (GOx) derived from *Aspergillus niger* has been thoroughly investigated, despite its occurrence in insects, larvae, and other fungi [17]. It is typically produced in microorganisms of the genera *Aspergillus* and *Penicillium* [18]. GOx (beta-D-glucose: oxygen-1-oxidoreductase, EC 1.1.3.4) is an enzyme belonging to the oxidoreductase family that catalyzes the oxidation of β-D-glucose into D-gluconolactone, using oxygen as an electron acceptor and releasing hydrogen peroxide (H_2_O_2_) [17,19]. It is a homodimeric glycoprotein with two identical 80 kDa subunits that is specific to the β-anomer of D-glucose but that can oxidize a variety of sugars (D-galactose, D-mannose, 2-deoxy-D-glucose, and D-xylose) at slower rates [20,21]. GOx follows a Ping-Pong Bi-Bi mechanism, composed of a reductive half-reaction for the oxidation of *β*-d-glucose and an oxidative half-reaction in which O_2_ is reduced [19]. During the reductive half-reaction, flavin adenine dinucleotide (FAD) is reduced to FADH_2_, while GOx catalyzes the production of δ-gluconolactone, which is later turned non-enzymatically to gluconic acid [22]. During the oxidative half-reaction, two protons and two electrons are transferred from FADH_2_ to O_2_ through two singlet electron transfer steps, producing H_2_O_2_ and returning GOx into its oxidized state [21]. GOx has surged as a starvation therapy for cancer due to the simultaneous consumption and depletion of oxygen and glucose, given the tumor’s susceptibility to variations in intracellular glucose levels [23,24]. Moreover, the presence of H_2_O_2_ may increase oxidative stress, inducing cell death due to an elevated amount of reactive oxygen species (ROS) [25]. However, glucose oxidase’s application in cancer therapy is restricted due to its immunogenicity, instability, short half-life in vivo, and systemic toxicity [23,26,27].

Nanoparticles (NPs) are generally defined as particles having at least one dimension in the size range of 1–100 nm and are characterised by unique attributes such as a high surface-area-to-volume ratio, tunable stability, and the potential for enhanced targeting and cellular uptake [28,29,30,31,32]. However, in some contexts, particularly in pharmaceutical sciences and nanomedicine, particles up to 1000 nm (1 μm) are loosely referred to as nanoparticles because they share certain colloidal behaviors and biological interactions with sub-100 nm particles [33]. The ease of functionalization, penetration abilities, enhanced drug loading, improved delivery, and extended retention inside its target have made NPs ideal for serving as drug delivery systems (DDS) [34,35,36]. Additionally, they can increase drug safety and efficacy by enhancing drug stability, bioavailability, and half-life, promoting across-membrane transport, controlling drug release, and improving the rate of uptake by target cells [28,37,38,39]. NPs have surged as an alternative strategy to conventional cancer therapies to reduce systemic toxicity by targeted, controlled and sustained drug administration [38]. Other advantages include enhanced pharmacokinetics, protection of healthy cells from cytotoxicity caused by the drug, accumulation of tumor tissues, and diminished drug resistance [40]. Mesoporous silica nanoparticles (MSNs) are porous structures with pore sizes from approximate 2 to 50 nm that are commonly used for drug delivery in cancer therapy due to their excellent biocompatibility, extended bloodstream circulation times, targeted drug delivery, negligible side effects on healthy tissues, high pore loading capacity, and easy functionalization [41,42,43]. In this study, the biogenic MSNs used were obtained from the local Mexican plant *Equisetum myriochaetum*, as reported earlier by our group [44]. These nanoparticles exhibit high biocompatibility and a wide range of mesopores, making them suitable for surface modifications and for encapsulating various phytochemicals, drugs, and enzymes. Furthermore, the high yield of silica from this plant species could be advantageous for scaling up the production of the prepared therapeutic products.

Recent studies have highlighted the importance of employing effective enzyme immobilization strategies to enhance catalytic efficiency and stability. Different immobilization chemistries, such as carbodiimide coupling, thiol-maleimide reactions, or click chemistry-based approaches, have been explored to achieve optimal enzyme orientation, preserve active sites, and improve reusability, thereby increasing the therapeutic efficacy of enzyme-based treatments [45]. Moreover, targeted therapy using ligand-functionalized nanoparticles enables specific delivery of therapeutic agents to cancer cells, sparing healthy tissues and improving treatment outcomes. Functionalizing MSNs with targeting moieties such as antibodies, peptides, or small molecules enhances cellular uptake and tumor accumulation, representing a promising strategy for precision oncology [46].

GOx-based nanosystems have been developed to enhance stability, tumor targeting, and therapeutic efficacy via the incorporation of GOx into metal–organic frameworks, polymeric NPs, and microspheres [47,48]. For instance, GOX-loaded Fe-based metal organic frameworks (Fe-MOFs) have exhibited antitumor activity in ovarian carcinoma [49], and co-delivery systems using arsenic trioxide and GOx enhanced uptake and therapeutic effects in SKOV3 cells [50]. In breast cancer models, GOx combined with curcumin or metformin in nanocarriers promoted ROS generation and triggered apoptosis pathways, improving efficacy while reducing toxicity on healthy cells [51,52]. Additionally, GOx-based systems have shown potential in multimodal therapies, such as in the incorporation of photothermal and chemodynamic components to achieve high cytotoxicity with minimal systemic effects [53]. These findings provide a strong rationale for the further exploration of GOx-based nanosystems in cancer.

Thus, this research aims to study the therapeutic potential of glucose oxidase-loaded biogenic MSNs in ovarian cancer cell lines. This investigation represents the first endeavor to investigate the anticancer benefits of immobilizing glucose oxidase on biogenic MSNs in OC cells. The expression levels of proto-onco and tumor suppressor genes, including *BCL-2*, *CTNNB1*, *TP53*, and *WNT1*, were studied to better understand the molecular effects of this nanoformulation.

## 2. Results and Discussion

### 2.1. Extraction of Biogenic MSNs and Immobilization of GOx

Biogenic MSNs were obtained via acid digestion of the *Equisetum myriochaetum* plant, commonly referred to as Mexican giant horsetail. This procedure yielded 11.74% of MSNs in a white powdered form. Previous research has demonstrated a threefold increase in yield when using *E. equisetum* for silica extraction through acid digestion, as compared to other *Equisetum* species (i.e., *E. arvense*) [44]. Biogenic MSN extraction from *E. myriochaetum* has been documented to have yields of 16.65% white powder [54]. Silicon (Si) concentrations in soils can range from less than 1% to 45% dry weight, whereas Si concentrations in plants can vary between 0.1% and 10% [55]. Thus, variations in yield may be attributed to differences in the concentration of silicic acid in the soil from which the plant came, as *Equisetum* spp. may absorb and accumulate silica in their tissues [56].

**Figure 1:**
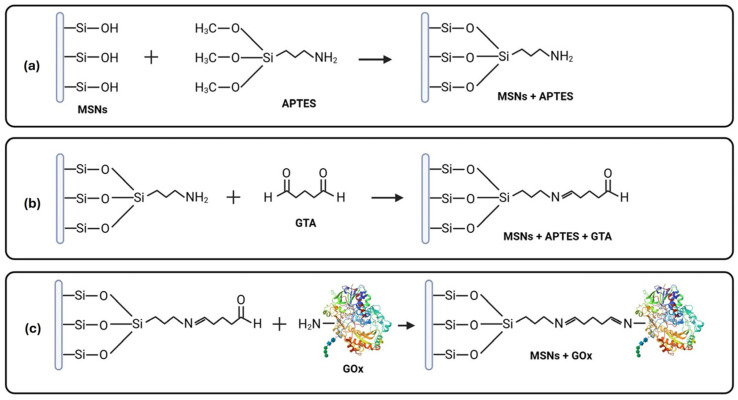
Schematic representation illustrating the process of biogenic MSN functionalization and immobilization with glucose oxidase (GOx). (**a**) Silanization process of MSNs with 3-aminopropyltriethoxysilane (APTES); (**b**) addition of aldehyde groups with glutaraldehyde (GTA) linker; (**c**) covalent binding of GOx to functionalized MSNs.

The immobilization process commenced with the silanization of the biogenic MSNs using APTES, a prevalent organosilane for surface modification (Figure 1). APTES interacts with silanol groups present in porous silicon surfaces, creating a uniform monolayer and providing amine termini that can engage with functional groups present in enzymes [57,58,59,60]. Silanization enhances enzyme orientation during immobilization, and amino functional modification with APTES has demonstrated superior efficacy in bio-receptor immobilization on silica surfaces compared to epoxy group modification [60,61]. This work employed post-deposition treatment following APTES modification, wherein biogenic MSNs were subjected to baking at 110 °C for 30 min. Variations in the temperatures and duration of this method have been reported to fortify and densify layers of aminopropyl silane, as thermal energy promotes horizontal polymerization of the self-assembled monolayer (SAM) [62,63]. Subsequently, GTA served as a linker to convert the amine functionality of APTES into aldehyde functional groups, enabling reactions with amine groups present in enzymes [59]. The use of GTA confers heightened stability between the enzyme and the carrier, as improved enzyme accommodation has been reported following its use [60,64]. This approach resulted in 49.24% of GOx being immobilized within the MSNs.

### 2.2. Characterization of the MSNs Pre- and Post-Immobilization

**Figure 2:**
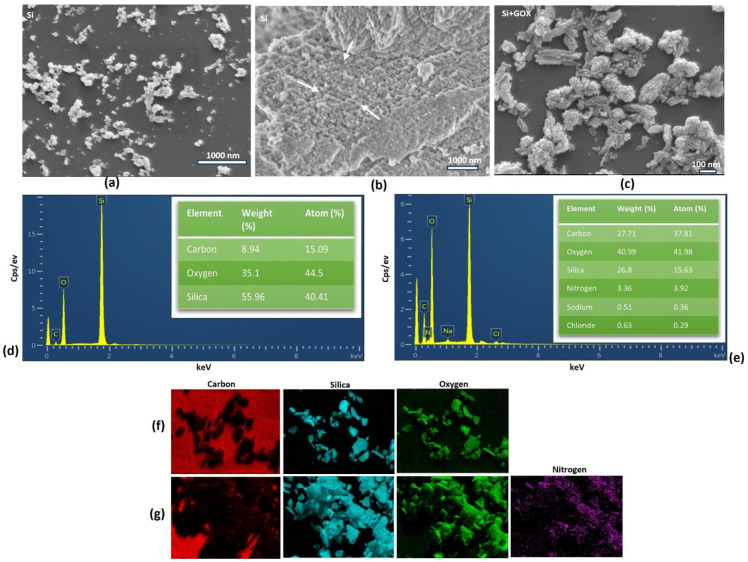
Scanning electron microscopy (SEM) of MSNs pre- and post-immobilization (with different magnifications). (**a**) Top view of biogenic MSNs; (**b**) surface view of biogenic MSNs (a few pores are indicated with arrows); (**c**) top view of GOX-immobilized MSNs; (**d**) energy-dispersive X-ray (EDX) spectroscopy of biogenic MSNs with accompanying table showing element percentages; (**e**) EDX spectroscopy of GOX-immobilized MSNs with element percentage table; (**f**) EDX-mapping of biogenic MSNs; (**g**) EDX-mapping of GOX-immobilized MSNs.

SEM analysis was performed to determine the topography and morphology of the biogenic MSNs [65]. A non-uniform morphology and agglomeration were observed, while the sample’s porosity is not discernible from this image (Figure 2a,b). While the GOx-immobilized MSNs showed a bigger particle size (Figure 2c). The sample’s elemental composition was analyzed using SEM coupled with energy-dispersive X-ray spectroscopy (EDX) [66]. EDX analysis indicated the presence of oxygen (O), silicon (Si), and carbon (C) in the free MSNs, listed in decreasing order of concentration (Figure 2d). The presence of nitrogen (N) was detected in the nanoformulation, attributed to the binding of the MSNs with APTES or GOx. The increase in carbon in this sample may be attributed to the incorporation of APTES, GTA, or the GOX enzyme (Figure 2e). Additionally, negligible amounts of sodium were also detected, which could originate from the buffers used during the protocol, and traces of chloride may have come from the commercial glutaraldehyde solutions, as they often contain chloride as a stabilizing agent. These results were validated by mapping, which confirmed the presence of nitrogen post-immobilization and provided the distribution of the constituent elements in the sample (Figure 2f,g). The diminished light intensity may suggest that N is present in lesser quantities relative to the other elements.

**Figure 3:**
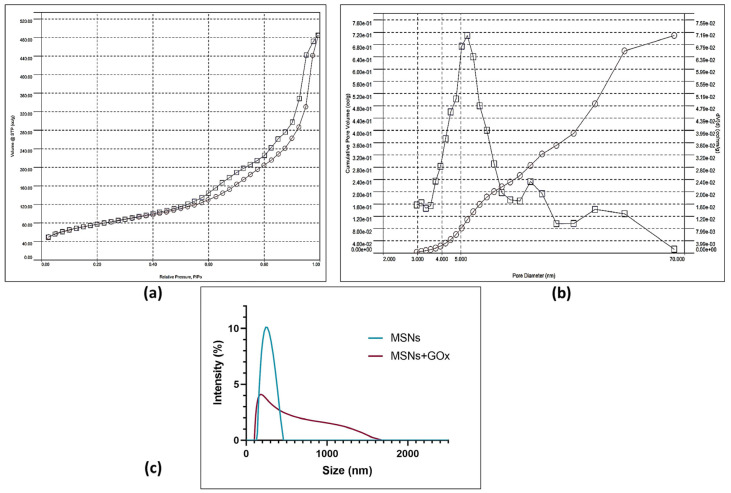
Characterization results. (**a**) Nitrogen adsorption/desorption isotherm of MSNs; (**b**) pore distribution of MSNs; (**c**) particle size distribution of free and immobilized MSNs.

The N_2_ adsorption–desorption isotherm and the related BJH pore size distribution are shown in Figure 3a,b. A type IV isotherm is identified, which is characteristic of mesoporous solids [67]. The IUPAC classification indicates that the sample exhibits a type H3 hysteresis, generally associated with solids having a wide pore size distribution [68]. The BET-specific surface area is 254.153 m^2^/g, with an average pore diameter of 5.397 nm and a pore volume of 0.71 cc/g (Table 1), consistent with the expected pore volumes ranging from 0.5 to 3 cm^3^/g [69]. Additionally, our silica has a pore diameter ranging from 3 nm to 62 nm, with many pores larger than 9 nm, making it suitable for accommodating the GOx enzyme, which has dimensions of 6.0 nm × 5.2 nm × 3.7 nm [70], allowing immobilization both on the surface and within the pores (Figure 3b). Elevated specific surface areas and pore volumes are advantageous for loading high payloads and enabling prolonged drug release [71].

The present study determined the average particle size of the biogenic MSNs before and after immobilization, considering the significance of NP size for cellular absorption and tumor infiltration [72] (Figure 3c). Ultrasonic treatment was employed for size refinement post-extraction, as it effectively facilitates size reduction, dispersion, de-agglomeration, and reduction of amalgamation of NPs [73,74]. Free MSNs exhibited a size of 240 nm (within the 50–300 nm range) and a polydispersity index of 16.18%, suitable for cellular uptake by endocytosis [43]. Moreover, NPs up to 200 nm demonstrate improved retention in tissue, resulting in heightened accumulation advantageous for passive targeting of most solid tumors through the enhanced permeability and retention (EPR) effect [75]. The nanoformulation exhibited a wider polydispersity index of 40.20%, attributable to the linkage with APTES, GTA, or GOx. Although the highest point for the nanoformulation can be observed at 174 nm, a broad distribution is present, suggesting sizes from 107 nm and 1550 nm. The variation and increase in size may be explained by the addition of molecules such as APTES, GTA, and GOx.

**Figure 4:**
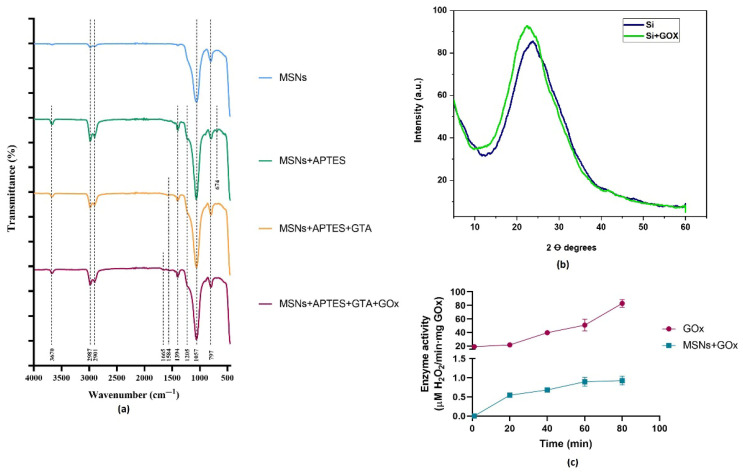
(**a**) Fourier transform infrared spectroscopy (FTIR) of biogenic MSNs alone, after silanization with APTES, functionalization with GTA, and immobilization with GOx; (**b**) X-ray diffraction (XRD) of free and immobilized MSNs; (**c**) enzymatic activity of free and immobilized GOx.

The mean zeta potential for the free MSNs was found to be −30.8 mV in distilled water at pH 6.2 at 25 °C, indicating a strong anionic charge [76]. Intriguingly, nanocarriers with a negative surface charge have been associated with favorable uptake, prolonged blood circulation, and enhanced distribution within tumors [77]. Additionally, MSNs exhibiting zeta potentials below −30 mV are deemed stable [78]. The mean zeta potential for the nanoformulation was 1.78 mV, indicating a neutral charge [76]. This alteration in zeta potential may result from the incorporation of APTES, GTA, or GOx. To verify the proper immobilization of GOx, FTIR spectra (450 to 4000 cm^−1^) were acquired for the biogenic MSNs alone, post-silanization with APTES, post-functionalization with GTA, and of the nanoformulation (Figure 4a). The spectra of all samples exhibited similarity, with the appearance of some additional peaks upon functionalization. The presence of silica was validated by the prominent peak observed at 1057 cm^−1^, associated with the stretching of SiO_2_ bonds [79,80,81]. All samples display bands at 2987 and 2901 cm^−1^, indicating the existence of CH_2_ asymmetric and symmetric stretching vibrations and C-H stretching vibrations, respectively, along with a band at 797 cm^−1^ linked to the asymmetric and symmetric stretching vibration of Si-O-Si bonds [82,83,84,85,86]. Following silanization with APTES, novel bands emerged at 3670, 1205, and 674 cm^−1^, corresponding to OH stretching vibration from water absorption, C-O vibrations, and Si-C bond vibrations, respectively [82,86,87]. However, no novel bands emerged upon functionalization with GTA. Lastly, the presence of a minor amide band at 1642 cm^−1^ in the nanoformulation suggests the immobilization of GOx [84,88].

XRD analysis was performed on both free and immobilized MSNs, as illustrated in Figure 4b. The broad bands with the highest peaks at 2θ = 22° and 2θ = 24° for immobilized and free MSNs, respectively, indicate the amorphous nature of the MSNs [89,90,91]. It has been established that humps with widths ranging from 15 to 30° from a 2θ Bragg angle indicate the existence of amorphous silica [92]. In contrast to the broad peaks observed in amorphous silica, crystalline structures exhibit sharp peaks at approximately 21°, 40°, 50°, 60°, and 68° [93,94]. The obtained amorphous silica and the prepared nanoformulation are suitable for use in biomedical applications [95]. In comparison to their crystalline counterparts, amorphous silica can enhance the biodegradation rate, which is an important factor for cytotoxicity [71].

To compare the activity of the same amount of GOx, calculations were performed considering the enzyme immobilized in the nanoformulation. The results showed an increase in activity over time for both systems, with the nanoformulation reaching a plateau after 60 min (Figure 4c). A decrease in activity was observed for the immobilized GOx, with the free enzyme showing an activity of 0.0987 ± 0.012 U, while the immobilized form had an activity of 0.0018 ± 0.0006 U, corresponding to specific activities of 49.36 ± 6.022 and 0.885 ± 0.31 U/mg, respectively. Therefore, the free enzyme’s activity was 55.75 times higher than that of the nanoformulation, indicating an activity retention of 1.79%. Since our silica nps exhibit a range of pore sizes, enzymes immobilized on the surface may behave differently from those that diffuse deeper into the pores. The reduced activity of immobilized GOx could be attributed to several factors associated with the immobilization process, including pore diffusion resistance, reduced substrate mass transfer, blockage of the enzyme’s active sites, and restricted enzyme mobility. Additionally, the loss of activity may occur if substrate diffusion into the nanoparticles is slower than the enzyme’s catalytic turnover rate, resulting in enzymes located deeper within the pores receiving less substrate compared to those closer to the surface [96,97]. However, studies have shown that enzyme activity is preserved by 97% after immobilizing GOx and catalase within mesoporous silica spheres (MSS) [98].

### 2.3. Cell Viability Assays of SKOV3 and HEK-293 Cells

**Figure 5:**
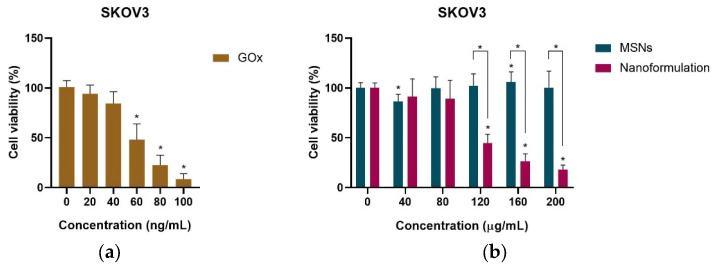
Cell viability assay on SKOV3 cells (**a**) after treatment with free Gox and (**b**) after treatment with MSNs and the nanoformulation. * *p* < 0.05.

Cell viability was evaluated 24 h post-application of biogenic MSNs, free GOx and the nanoformulation on SKOV3 cells. The results indicate a concentration-dependent reduction in cell viability with the application of free or immobilized GOx (Figure 5a). The IC_50_ values were determined to be 60.77 ng/mL for free GOx and 111.6 µg/mL for the nanoformulation (Table 2). These results indicate that the free enzyme has a strength 46.99 times greater than that of its immobilized counterpart, corroborating the findings from the enzymatic activity assay. Although approximately 2.8 µg of immobilized GOx is present on 111.6 µg of silica, multiple reasons could be proposed for its lower performance in comparison to free GOx. One of the primary reasons could be steric hindrance to the passage of glucose through the pores of the silica nps, which is required for the generation of ROS by GOx, a necessary phenomenon for effective cytotoxicity. Apart from that, performance could also be impacted if the active site is blocked due to improper orientation or if distortion of the enzyme’s structure has occurred during immobilization. However, a significant impact of the nanoformulation was detected at a concentration of 120 µg/mL, with minimal cell viability being observed at 18.06% for 200 µg/mL.

Meanwhile, the biocompatibility of biogenic MSNs was assessed following treatment with varying concentrations on SKOV3 cells (Figure 5b). A notable effect on cell viability was observed at doses of 40 µg/mL and 160 µg/mL, whilst the remaining values did not influence cell survival, indicating biocompatibility. The nanoformulation exhibited a significant cytotoxic effect, with a minimum cell viability of 18.06% achieved at 200 µg/mL. A minimum cell viability of 85.6% was recorded at a concentration of 40 µg/mL of biogenic MSNs, validating findings that demonstrated good biocompatibility of MSNs, which exhibited a cell viability of 90% [97]. The cytotoxicity of biogenic MSNs and the nanoformulation was compared, demonstrating a significant difference in impact between 120 µg/mL and 200 µg/mL. This suggests that at lower doses, the cytotoxic impact may not be attributable to the action of GOx.

**Figure 6:**
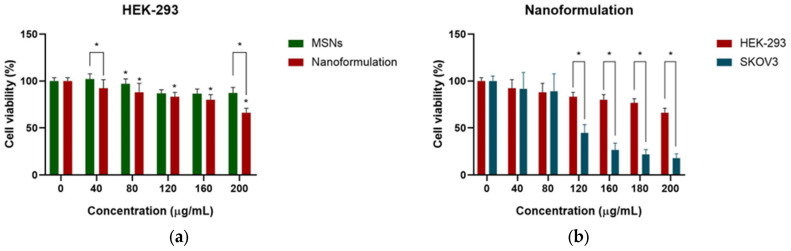
Cell viability assay on HEK-293 cells (**a**) after treatment with MSNs and the nanoformulation; (**b**) comparison with SKOV3 cells after treatment with the nanoformulation. * *p* < 0.05.

Due to their low cost, simplicity of manipulation, and reproducibility, immortalized cells are utilized to examine the toxicity of nanomaterials [99,100]. The HEK-293 cell line is one of the most often used as a control, normal, or comparator cell line [101]. Although normal ovarian cells can be sustained in vitro for a duration of up to 8 weeks, a decline in specific markers occurs, followed by apoptosis or senescence, making it difficult to be maintained as a continuous culture [102]; moreover, it is challenging to obtain healthy adult ovarian cell lines. Thus, the impact of biogenic MSNs and the nanoformulation was evaluated on a healthy cell line, HEK-293, to demonstrate their impact on healthy tissues (Figure 6a). The results indicate the biocompatibility of biogenic MSNs with HEK-293 cells, exhibiting a maximum cell viability of 86.38%, similar to the findings in SKOV3 cells. Meanwhile, the nanoformulation exhibited a concentration-dependent effect. Cell viability of 66.26% at a concentration of 200 µg/mL was registered. The comparison of the cytotoxic effects of free and immobilized MSNs indicates a significant reduction in cell viability exclusively at concentrations of 40 µg/mL and 200 µg/mL. The cytotoxic effects of the nanoformulation on SKOV3 and HEK-293 were compared, revealing a significant difference in cell viability at concentrations of 120 µg/mL and 200 µg/mL (Figure 6b). The nanoformulation exhibited analogous behaviors at lower concentrations; however, a marked increase in cytotoxicity was observed in cancer cells at elevated doses. An increased cytotoxicity and no systemic toxicity were observed on melanoma and triple-negative breast cancer cells after treatment with GOx-coated copper sulfide NP nanocomposites [103]. Furthermore, higher cytotoxicity against prostate cancer cells was noted following treatment with a combination of a GOx, poly-L-lysine-grafted-polyethylene glycol, and an anti-prostatic specific membrane system, in contrast to GOx alone, resulting in 60% cell viability post-treatment [104].

Despite identical treatments of the SKOV3 and HEK-293 cell lines, the noted selectivity in cytotoxicity may stem from intrinsic physiological differences between the cells. The Warburg effect states that glucose uptake increases significantly to facilitate the growth, survival, proliferation, and sustained maintenance of cancer cells [105]. Cancer cells exhibit a heightened susceptibility to glucose deprivation relative to normal ones, frequently leading to apoptosis, reduced proliferation, growth inhibition, cell cycle arrest, and autophagy [106,107]. Cancer cells have elevated amounts of ROS in comparison to normal cells [108]. If the elevation of ROS surpasses a specific threshold detrimental to cellular viability, ROS may induce cytotoxic effects, resulting in the demise of malignant cells and thereby inhibiting cancer progression [109]. This susceptibility to glucose deprivation and oxidative stress may account for the increased cytotoxicity observed in SKOV3 cells relative to HEK-293 cells.

### 2.4. Differential Gene Expression Analysis by qPCR

Cancer is a complex progressive disease marked by the impairment of multiple systems, including DNA repair, apoptosis, and immune responses [110]. The majority of cancers result from the combination of multiple genetic alterations occurring during tumor progression rather than from a singular oncogenic driver [111]. Therefore, this study aimed to explore the differential expression of genes associated with carcinogenesis (including *BCL-2*, *CTNNB1*, *TP53*, and *WNT1*) in SKOV3 cells after being treated with GOx-loaded biogenic MSNs compared to an untreated control group.

**Figure 7:**
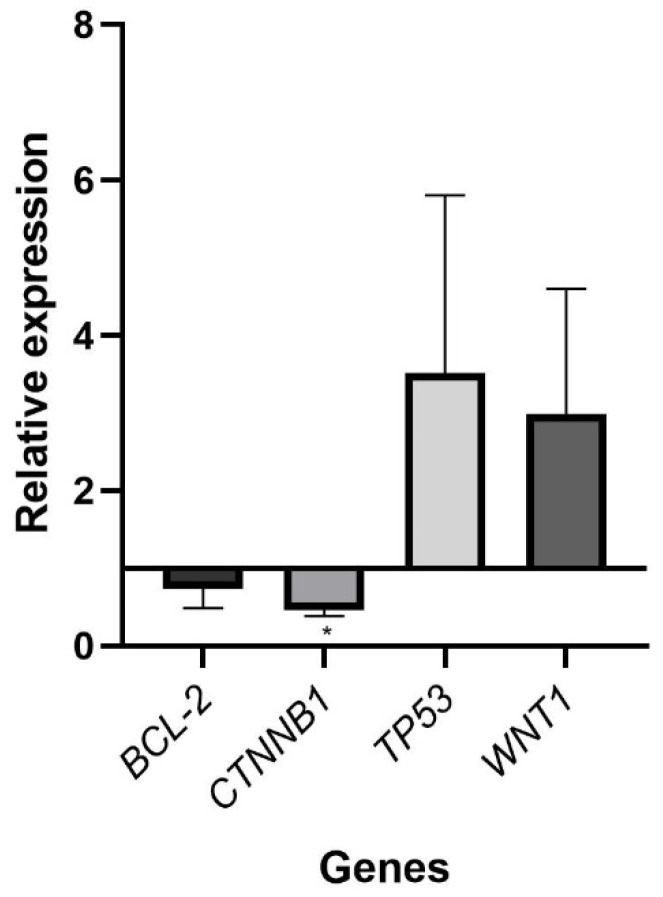
Expression profile of cancer-related genes (*BCL-2*, *CTNNB1*, *TP53* and *WNT1*) after treatment with the nanoformulation (111.6 µg/mL) for 24 h. *GAPDH* was used as an endogenous control for normalization. Each bar graph depicts the mean values of relative fold change ± standard deviation of the biological replicates (* *p* < 0.05).

The oncogene β-catenin (*CTNNB1*) exhibited a significant downregulation (*p* < 0.05) in the treated samples (Figure 7), aligning with its non-significant downregulation following doxorubicin treatment of SKOV3 cells [112]. *CTNNB1* is implicated in the pathogenesis of most cancers, contributing to tumor immune infiltration [113]. OC metastasis and therapeutic resistance are linked to the Wnt signaling pathway, in which *CTNNB1* plays a significant role [114,115,116]. Given the frequent occurrence of *CTNNB1* mutations in ovarian endometrioid carcinoma, particularly in cisplatin-resistant OC, *CTNNB1* may hold clinical significance in ovarian carcinoma [117,118]. Although information regarding the differential expression of *CTNNB1* in OC is scarce, *CTNNB1* has been associated with other malignancies. In this context, PS341 resulted in the downregulation of *CTNNB1* in hepatocellular carcinoma and colorectal cancer, inducing apoptosis, cell cycle arrest, and inhibiting migration, invasion, and tumorigenesis in these malignancies [119]. The knockdown of *CTNNB1* resulted in an induction of apoptosis and the suppression of cell proliferation, migration, and invasion in renal cell carcinoma cell lines [120]. Similarly, its knockdown was observed to inhibit the proliferation of human adrenocortical carcinoma cells [121].

*TP53* is the most frequently mutated tumor-suppressor gene in human malignancies, occurring in 96% of high-grade serous ovarian cancer (HGSOC) [122]. Wild-type *TP53* facilitates cell-cycle arrest and regulates transcription genes linked to senescence and apoptosis; hence, its loss of function promotes carcinogenesis through the bypass of growth arrest, allowing continued proliferation [123,124]. Despite the absence of significant findings, a trend indicating upregulation of *TP53* in SKOV3 cells following treatment with the nanoformulation was observed. Numerous studies have demonstrated an upregulation of *TP53* in SKOV3 cells, including following treatment with gold NPs loaded with linalool; modified with glutathione and conjugated with the CALNN peptide; and modified with graphene oxide-silver NPs and trichostatin (both individually or in combination or after the application of salidroside) [125,126,127].

The BCL-2 family of proteins, which includes pro-apoptotic proteins (BAX, BAK, and BOK), survival proteins (BCL-2, BCL-XL, MCL-1), and pro-apoptotic BH3-only proteins (BIM, BID, BAD, HRK), regulates apoptotic cell death [8,9]. Cancer cells block apoptosis by upregulating anti-apoptotic BCL-2 proteins, which inhibit BAX, BAK, and BH3-only proteins [8]. The expression of *BCL-2* and *BAX* has been identified as a prognostic factor in ovarian cancer due to their predictive significance in OC patients [10,128]. Although an anticipated downregulation trend of the anti-apoptotic *BCL-2* gene was observed in this study, no significant results were found. A decrease in *BCL-2* expression was noted following treatment with alantolactone, cisplatin, and zinc oxide NPs as single agents, in double and triple combinations [129], and after treatment with metformin NPs in SKOV3 cells [130].

The Wnt/β-catenin pathway governs metastasis promotion, cancer proliferation, chemoresistance, immune response, and enhancement of tumor angiogenesis [131]. This pathway has been identified as a contributor to the pathophysiology of gynecological cancers, including OC, due to its role in the tumor progression, recurrence, and chemoresistance of these malignancies [132]. Canonical Wnt signaling is activated by *WNT1*, *WNT2*, *WNT3*, *WNT3a*, and *WNT8b*, among others, with *WNT1* levels significantly elevated in OC relative to normal ovaries [11,133]. An unforeseen increase in *WNT1* expression was noted following treatment with the nanoformulation; however, the results were not statistically significant within a 95% confidence interval. Evidence indicates that the Wnt/β-catenin pathway has a role in the preservation of stem-like properties and drug resistance of HGSOC [134]. Thus, the unexpected upregulation of *WNT1* following therapy with the nanoformulation may result from the persistence of resistant cell populations after treatment.

### 2.5. Future Perspectives

Despite the promising findings in this study, it would be beneficial to integrate diverse ways to enhance the efficacy of GOx as a therapeutic agent for OC while further mitigating its impact on healthy cells, which can be accomplished via targeted therapies. Active targeting enhances the retention, penetration, and uptake of pharmaceuticals at tumor sites by conjugating targeting ligands—including antibodies, peptides, small molecules, and aptamers—to the surface of NPs to identify cancer cell receptors [135]. Moreover, pH-responsive polyethylene glycol (PEG) effectively conceals targeting ligands at physiological pH, revealing them in acidic tumor conditions [136]. For instance, cell surface receptors such as human epidermal growth factor 2 (HER2), folic acid (FA) receptors, the cluster of differentiation 44 (CD44), and vascular endothelial growth factor (VEGF) are used for recognition since they are significantly overexpressed in OC cancer cells, aiming for increased internalization [137]. The utilization of doxorubicin (DOX) delivery systems with FA-modified epigallocatechin gallate or FA-modified poly(d, l-lactide-co-glycolide) (PLGA) NPs significantly enhanced cellular absorption, cytotoxicity, and tumor volume inhibition in SKOV3 cells [138,139]. Lipid NP therapy with mRNA encoding HER2-CD3-Fc bispecific antibodies inhibited the growth of HER2-positive tumors, demonstrating an antitumor effect in a murine xenograft model [140]. Concurrently, the anti-HER2 antibody trastuzumab conjugated to the PLK1-targeted agent volasertib exhibited increased uptake and antitumor efficacy in HER2-overexpressing SKOV3 cells [141]. Since hyaluronic acid is the primary ligand for CD44 receptors, HA-coated polymeric paclitaxel NPs accumulated in CD44-expressing SKOV3, resulting in reduced cell proliferation, enhanced cytotoxicity, and increased apoptosis [142].

Although the nanoformulation displayed a promising anti-cancer effect against OC cells in this study, there is potential for enhanced therapy efficacy through combination with other drugs. Several studies have been conducted utilizing combined therapy with GOx for the treatment of various cancers. In this sense, the combination of GOx, DOX, oxygen, and cancer cell membrane (CCM) within hollow mesoporous organosilica NPs (HMONs) leveraged their starvation, apoptosis, and increased cellular uptake properties for the therapy of hepatocellular carcinoma [143]. The combined action of GOx and DOX was used in pH-responsive FA-targeting iron oxide core–shell magnetic MSNs, demonstrating increased cytotoxicity, apoptosis induction, and tumor growth inhibition in pancreatic mouse models while maintaining favorable biosafety [144]. The amalgamation of GOx with tirapazamine (TPZ) in bilirubin-functionalized HMONs augmented cancer starvation and safeguarded healthy tissue [145]. A starvation-induced synergism, along with enhanced chemodynamic treatment, demonstrated significant therapeutic efficacy in breast cancer cells via GOx-grafted MSNs and Fe_3_O_4_ NPs [146].

Certain approaches can be implemented to improve enzyme-immobilization performance [147]. The modification of particle size and pore structure can enhance immobilization, increase enzyme loading, and improve enzyme stability [148]. Larger pore sizes may provide inadequate protection for enzymes, whereas smaller pore sizes may be excessively constrictive for optimal enzyme functionality [149]. Furthermore, surface charge may influence enzyme immobilization and activity regulation. In this context, positively charged NPs attained the largest immobilization percentage of HRP, although this resulted in a considerable loss of activity, while near-neutral and negatively charged MSNs ensured stability and activity retention [150].

## 3. Materials and Methods

### 3.1. Materials

*Equisetum myriochaetum* plant, distilled water, nitric acid (HNO_3_), sulfuric acid (H_2_SO_4_), dimethyl sulfoxide (DMSO), and phenol were obtained from Karal (Guanajuato, Mexico). Bradford Reagent, toluene, 3-aminopropyltriethoxysilane (APTES), glutaraldehyde solution (GTA, 25% (*v*/*v*)), 4-Aminoantipyrine, phosphate-buffered saline (PBS), and glucose oxidase from *Aspergillus niger* were purchased from Sigma-Aldrich (St. Louis, MO, USA). Horseradish peroxidase was provided by GoldBio (St. Louis, MO, USA). Anhydrous D-glucose was purchased from Macron Fine Chemicals, Avantor (Center Valley, PA, USA). Fetal bovine serum, Roswell Park Memorial Institute 1640 (RPMI-1640), Dulbecco’s Modified Eagle Medium (DMEM), and antibiotic–antimycotic solution (100X, 10,000 units/mL of penicillin, 10,000 μg/mL of streptomycin, and 25 μg/mL of Gibco Amphotericin B) were provided by Gibco, Thermo Fisher Scientific (Waltham, MA, USA). Human ovarian adenocarcinoma cell line (SKOV3) and Human Embryonic Kidney (HEK-293) were obtained from the American Type Culture Collection (ATCC). We further utilized 3-(4,5-dimethylthiazol2-yl)-2,5-diphenyltetrazolium bromide (MTT) (Invitrogen, Thermo Fisher Scientific, Waltham, MA, USA), the miRNeasy Mini Kit (Qiagen, Hilden, Germany), the Mir-X miRNA First-Strand Synthesis Kit (Tokyo, Japan), and the Mir-X miRNA qRT-PCR SYBR Kit (Tokyo, Japan). All the primers (*BCL-2*, *CTNNB1*, *GAPDH*, *TP53*, and *WNT-1*) used were provided by Merck (Darmstadt, Germany).

### 3.2. Biogenic MSNs Extraction

In the current study, biogenic MSNs were obtained from the *Equisetum myriochaetum* plant, with slight modifications to the protocol described by Ochoa-Sánchez and collaborators (2024) [54]. Plant material was recollected, sun-dried for 72 h, separated into stems and branches, and individually ground into a fine powder. For the extraction, 50 g of stem powder was subjected to acid digestion using 2 L of a concentrated HNO_3_/H_2_SO_4_ (4:1) solution and stirred on a hot plate at 70 °C for 24 h until the appearance of a white precipitate. The acids were removed, and a new solution of HNO_3_/H_2_SO_4_ (4:1) was introduced, maintained under the same conditions for 24 h. The white precipitate was left to sediment, the acids were removed, and 1 L of distilled water was introduced and left to settle overnight. The recovered precipitate was washed with abundant distilled water until reaching a neutral pH. Subsequently, the sample was subjected to rounds of ultrasonication, in which 100 mg of sample was mixed with 30 mL of a 1:1 ethanol–water solution, ultrasonicated for 30 s at an amplitude of 100 A, and left to rest for 5 min, 5 times. The mixture was left to precipitate for 10 min, the supernatant was recovered, and the cycles were repeated with the precipitate until no precipitate was observed after 10 min of sedimentation. The sample was calcined at 650 °C for 5 h.

### 3.3. Glucose Oxidase Immobilization

Silanization was used for the immobilization of glucose oxidase from *Aspergillus niger* based on a previously described protocol [151] with several modifications. Thirty milliliters of a freshly prepared 2% (*v*/*v*) APTES solution in toluene was stirred for 20 min at room temperature. Then, 300 mg of biogenic MSN was added to the solution and sonicated for 30 min, followed by stirring for 2 h at room temperature. Anhydrous toluene was used to rinse the modified NPs, which were then baked at 110 °C for 30 min. The biogenic MSNs were washed with water 3 times, resuspended in 40 mL sodium acetate buffer solution (50 mM, pH 5.1) containing 1% GTA (25% (*v*/*v*)), and stirred for 1.5 h. The functionalized NPs were washed 3 times with sodium acetate buffer solution and recovered by centrifugation. A solution containing 16 mg of GOx in 38.4 mL of sodium acetate buffer was added to the NPs and left to stir in a cold room for 24 h. The supernatant was recovered, and the immobilized MSNs were washed 3 times with sodium acetate buffer. The supernatant was recovered for subsequent analysis. The nanoformulation was lyophilized for 36 h and stored at *−*20 °C.

### 3.4. Determination of Drug Encapsulation Efficiency

The encapsulation efficiency of GOx in the biogenic MSNs was obtained by the Bradford assay, with a bovine serum albumin (BSA) curve with concentrations from 0.1 to 1 mg/mL. The supernatants from the immobilization and the 3 washes were used as samples. In a 96-well plate, 5 μL of sample was loaded into separate wells in triplicate, followed by adding 250 μL of Bradford reagent that was previously brought to room temperature. The plate was shaken for 30 s and incubated for 10 min. The absorbance was measured at 595 nm with a Multiskan SkyHigh spectrophotometer (Thermo Fisher Scientific, Waltham, MA, USA). The net absorbance was obtained, and the drug encapsulation efficiency (DEE) was calculated with the following formula:

$$DEE=\left[\frac{Initial\;GOx\;amount-GOx\;amount\;in\;supernatant}{Initial\;GOx\;amount}\right]\times 100\%$$

### 3.5. Activity Assay

A colorimetric method was employed for the determination of free and immobilized GOx activity [152]. During this assay, GOx catalyzed the conversion of D-glucose to gluconic acid, releasing H_2_O_2_. In the presence of peroxidase, a second reaction occurs in which the H_2_O_2_ reacts with 4-amino antipyrine and phenol for the stoichiometric formation of a red quinoneimine dye complex that has a maximum absorption at 510 nm [151]. With a calibration curve, the amount of H_2_O_2_ produced by GOx was calculated from the UV absorbances of the samples. In this assay, 200 µL of a 10 µg/mL free GOx solution or of a 380 µg/mL immobilized GOx solution was mixed with 3.5 mL PBS (0.01 M, pH 7.0) and 800 µL of a 50 mM D-glucose solution in PBS for different times (0, 20, 40, 60, and 80 min). Subsequently, 2.5 mL of these solutions was mixed with 1 mL 4-AAP (20 mM), 1 mL phenol (20 mM), and 500 µL of an HRP solution (0.05 mg/mL), after which the color change was analyzed at 510 nm by a spectrophotometer (xMark Microplate Spectrophotometer, BioRad, Hercules, CA, USA). Similarly, the calibration curve was established using varying concentrations of H_2_O_2_ (0, 120, 240, 360, 480, 600, 720, and 840 µM) in 2.5 mL of PBS. Triplicates were used to determine the enzymatic activity. One unit (U) of activity was defined as the amount of enzyme that oxidized 1 µmol of substrate per minute at 25 °C and pH 7.0.

### 3.6. Biogenic MSNs Characterization Pre- and Post-Immobilization

Functional group identification was performed using a Fourier transform infrared spectrometer (FT-IR Spectrometer PerkinElmer, Waltham, MA, USA) set to a scanning range between 450 and 4000 cm*^−^*^1^. Dynamic light scattering (DLS) measurement was conducted to determine the size and zeta potential of the extracted silica as well as the prepared nanoformulation using the Litesizer DLS 500 (Anton Paar, Graz, Austria). Structural analysis was obtained by X-ray diffractometer (Ultima IV, Rigaku Corporation, Tokyo, Japan) using Cu-Kα radiation. The sample’s morphology and composition were characterized by scanning electron microscopy (SEM) using the Hitachi SU8230 cold field emission SEM (Hitachi, Tokyo, Japan) with an energy-dispersive X-ray (EDX) detector. The Brunauer–Emmett–Teller (BET) method, along with the Barret–Joyner–Halenda (BJH) method, was employed to determine pore size distribution.

### 3.7. Cell Culture

A human ovarian adenocarcinoma cell line (SKOV3) and a human embryonic kidney cell line (HEK-293) were employed in this study. SKOV3 was cultured in Roswell Park Memorial Institute 1640 (RPMI 1640) medium with L-glutamine, whereas HEK-293 was cultured in Dulbecco’s Modified Eagle Medium (DMEM) with high glucose. Both media were supplemented with 10% fetal bovine serum (FBS) and 1% antibiotic–antimycotic solution (100X, 10,000 units/mL of penicillin, 10,000 μg/mL of streptomycin, and 25 μg/mL of Gibco Amphotericin B). For the maintenance of desired confluency, cell cultures were kept in a 5% CO_2_ humidified incubator at 37 °C.

### 3.8. Cell Viability Assay

A cell viability assay in the presence of free GOx, free MSNs, and immobilized nanoformulation was performed with the 3-(4,5-dimethylthiazol-2-yl)-2,5-diphenyltetrazolium bromide (MTT) assay (Sigma-Aldrich). SKOV3 and HEK-293 cells were cultured in 96-well plates at a seeding density of 10,000 cells per well for 24 h at 37 °C and 5% CO_2_. After the removal of the medium, 200 *μL* of different concentrations of treatment in the medium were applied. Free GOx was studied in concentrations from 0–100 ng/mL on SKOV3 cells, while concentrations ranging from 0 to 200 μg/mL were used for free and immobilized MSNs in both SKOV3 and HEK-293 cells. After incubation for 24 h with their respective treatment, 300 μL of MTT (5 mg/mL) was added to each well, followed by incubation for 4 h. MTT was removed, formed formazan crystals were dissolved in 200 μL of DMSO, and the absorbance was measured at 570 nm with the Multiskan SkyHigh Microplate Spectrophotometer (Thermo Fisher Scientific). The cell viability was calculated with the following formula; subsequently, the IC_50_ dose was estimated.

$$Cell\;viability\left(\%\right)=\frac{{OD}_{treated\;cells}}{{OD}_{control\;cells}}\times 100\%$$

### 3.9. RNA Extraction, cDNA Synthesis, and Expression Analysis by RT-qPCR

For gene expression analysis, SKOV3 cells were initially seeded into T25 flasks and incubated until reaching 80% confluency. Then, the previously obtained IC_50_ dose (111.6 µg/mL) of the nanoformulation was applied to SKOV3 cells, while the medium was replaced in the control cells. They were left incubating for 24 h at 37 °C and 5% CO_2_. The isolation and purification of total RNA from control and treated cells were performed with the miRNeasy Mini Kit (Qiagen, Hilden, Germany) following the manufacturer’s instructions. The quality and concentration of the RNA were assessed with the NanoDrop One spectrophotometer (Thermo Scientific). The synthesis of cDNA was performed using the Mir-X miRNA First-Strand Synthesis Kit (Takara, Tokyo, Japan).

The qPCR analysis was conducted using reagents from the Mir-X miRNA qRT-PCR SYBR Kit (Takara, Tokyo, Japan) on a Step One Real-Time PCR System (Applied Biosystems, Foster City, CA, USA). Forward and reverse primers for cancer-related genes (*BCL-2*, *CTNNB1*, *TP53*, and *WNT1*) were used for the analysis of target gene expression. The primers used can be found in Table 3. The reactions, with a final volume of 12.5 µL, contained 1 × SYBR Advantage Premix, 1 × ROX dye, 0.2 µM of each primer (forward and reverse), and 1 µL of the first-strand cDNA. The parameter for qPCR reactions comprised an initial denaturation phase at 95 °C for 10 s, succeeded by 45 cycles of denaturation at 95 °C for 5 s, annealing at 55 °C for 15 s, and extension at 72° C for 15 s, concluding with a melting curve of 95 °C for 1 min, 55 °C for 30 s, and 95 °C for 30 s. All qPCR reactions were conducted in biological triplicates, with three technical replicates for both control and treatment cells. The delta-delta Ct method (2^−ΔΔCt^), with *GAPDH* as an endogenous normalization control, was utilized to quantify differential gene expression.

### 3.10. Statistical Analysis

Student’s *t*-test was utilized to assess the statistical significance between the groups of biological replicates, whilst a one-way ANOVA was employed to determine the statistical significance of varying treatment concentrations. A *p*-value of less than 0.05 was deemed statistically significant. These results are illustrated in the graphs as the mean value ± the standard error of the biological replicates.

## 4. Conclusions

Immobilization of GOx onto biogenic MSNs was accomplished using APTES as a silane coupling agent and GTA as a crosslinker, resulting in a GOx carrier system. This nanoformulation not only enhanced cytotoxic activity towards SKOV3 cells relative to healthy cells but also demonstrated a significant downregulation of the oncogene *CTNNB1*, suggesting a targeted molecular response. The novelty of this nanoformulation lies in the use of biogenic MSNs, which offer a biocompatible and biodegradable platform, and in the covalent immobilization strategy. Additionally, the selective cytotoxicity emphasizes the therapeutic potential of this system to minimize off-target effects. Overall, GOx-loaded biogenic MSNs present a promising strategy for OC treatment; however, further studies are warranted to elucidate the mechanisms by which GOx influences the amelioration of ovarian cancer.

## Figures and Tables

**Table 1:** BJH desorption summary.

Specific Surface Area (m^2^/g)	Average Pore Volume (cc/g)	Average Pore Diameter (nm)
254.153	0.710	5.397

**Table 2:** Comparison between free and immobilized GOx.

	Free GOx	Immobilized GOx
IC_50_	60.77 ng/mL	111.6 µg/mL
Enzymatic activity (U)	0.0987 ± 0.012	0.0018 ± 0.0006

**Table 3:** Primer sequences used for qPCR analysis.

Gene	Forward Primer (5′–3′)	Reverse Primer (5′–3′)
*BCL-2*	GCCTTCTTTGAGTTCGGTGG	GAAATCAAACAGAGGCCGCA
*CTNNB1*	TGAGGAGCAGCTTCAGTCCC	CTTGAGTAGCCATTGTCCACG
*GAPDH*	ACAGTTGCCATGTAGACC	TTGAGCACAGGGTACTTTA
*TP53*	ACCTATGGAAACTACTTCCTG	ACCATTGTTCAATATCGTCC
*WNT1*	CCCTAACCGGTGCGCCCTGGTGC	AGCGCCCAGAGCCCCATGGCCTG

## Data Availability

The data that supports the findings of this research are available from the corresponding author upon request.

## Abbreviations

The following abbreviations are used in this manuscript:

OC	Ovarian cancer
GOx	Glucose oxidase
MSNs	Mesoporous silica nanoparticles
DDS	Drug delivery systems
APTES	3-aminopropiltrietoxysilane
GTA	Glutaraldehyde
CTNNB1	Β-catenin
EOC	Epithelial ovarian cancer
H_2_O_2_	Hydrogen peroxide
FAD	Flavin adenine dinucleotide
ROS	Reactive oxygen species
NP	Nanoparticles
HNO_3_	Nitric acid
H_2_SO_4_	Sulfuric acid
DMSO	Dimethyl sulfoxide
PBS	Phosphate-buffered saline
RPMI	Roswell Park Memorial Institute 1640
DMEM	Dulbecco’s Modified Eagle Medium
SKOV3	Human ovarian adenocarcinoma
HEK-293	Human embryonic kidney
MTT	3-(4,5-dimethylthiazol2-yl)-2,5-diphenyltetrazolium bromide
BSA	Bovine serum albumin
DEE	Drug encapsulation efficiency
FTIR	Fourier transform infrared spectroscopy
DLS	Dynamic light scattering
XRD	X-ray diffractometer
SEM	Scanning electron microscopy
EDX	Energy-dispersive X-ray spectroscopy
BET	Brunauer–Emmett–Teller
BJH	Barret–Joyner–Halenda
FBS	Fetal bovine serum
SAM	Self-assembled monolayer
MSS	Mesoporous silica spheres
HGSOC	High-grade serous ovarian cancer
PEG	Polyethylene glycol
HER2	Human epidermal growth factor 2
FA	Folic acid
CD44	Cluster of differentiation 44
VEGF	Vascular endothelial growth factor
DOX	Doxorubicin
PLGA	Poly(d,l-lactide-co-glycolide)
FAK	Focal adhesion kinase
CCM	Cancer cell membrane
HMON	Hollow mesoporous organosilica nanoparticle
TPZ	Tirapazamine
